# Flexible modeling of headache frequency fluctuations in migraine with hidden Markov models

**DOI:** 10.1111/head.14782

**Published:** 2024-07-30

**Authors:** Gina M. Dumkrieger, Ryotaro Ishii, Peter J. Goadsby

**Affiliations:** ^1^ Mayo Clinic Phoenix Arizona USA; ^2^ Kyoto Prefectural University of Medicine Kyoto Japan; ^3^ National Institute for Health Research (NIHR) King's Clinical Research Facility King's College London London UK; ^4^ Department of Neurology University of California Los Angeles California USA

**Keywords:** headache, hidden Markov model, migraine

## Abstract

**Objective:**

To explore hidden Markov models (HMMs) as an approach for defining clinically meaningful headache‐frequency‐based groups in migraine.

**Background:**

Monthly headache frequency in patients with migraine is known to vary over time. This variation has not been completely characterized and is not well accounted for in the classification of individuals as having chronic or episodic migraine, a diagnosis with potentially significant impacts on the individual. This study investigated variation in reported headache frequency in a migraine population and proposed a model for classifying individuals by frequency while accounting for natural variation.

**Methods:**

The American Registry for Migraine Research (ARMR) was a longitudinal multisite study of United States adults with migraine. Study participants completed quarterly questionnaires and daily headache diaries. A series of HMMs were fit to monthly headache frequency data calculated from the diary data of ARMR.

**Results:**

Changes in monthly headache frequency tended to be small, with 47% of transitions resulting in a change of 0 or 1 day. A substantial portion (24%) of months reflected daily headache with individuals ever reporting daily headache likely to consistently report daily headache. An HMM with four states with mean monthly headache frequency emissions of 3.52 (95% Prediction Interval [PI] 0–8), 10.10 (95% PI 4–17), 20.29 (95% PI 12–28), and constant 28 days/month had the best fit of the models tested. Of sequential month‐to‐month headache frequency transitions, 12% were across the 15‐headache days chronic migraine cutoff. Under the HMM, 38.7% of those transitions involved a change in the HMM state, and the remaining 61.3% of the time, a change in chronic migraine classification was not accompanied by a change in the HMM state.

**Conclusion:**

A divide between the second and third states of this model aligns most strongly with the current episodic/chronic distinction, although there is a meaningful overlap between the states that supports the need for flexibility. An HMM has appealing properties for classifying individuals according to their headache frequency while accounting for natural variation in frequency. This empirically derived model may provide an informative classification approach that is more stable than the use of a single cutoff value.

## Plain Language Summary

A hidden Markov model is a complex statistical model that can group patients with migraine by headache frequency in a way that is more flexible than the current standard episodic versus chronic cutoff defined by the International Classification of Headache Disorders. The groups defined by this model appeared to differ in clinically meaningful measures and migraine characteristics. Such a model may be useful for defining groups for research and/or for differentiating meaningful changes in headache frequency from natural variation.

**Abbreviations**
AIC: Akaike information criterion
ARMR: American Registry for Migraine Research
CI: confidence interval
HMM: hidden Markov model
ICHD: International Classification of Headache Disorders

## INTRODUCTION

Monthly migraine frequency can have significant implications for the diagnosis and treatment of migraine and migraine subtypes and for the evaluation of migraine clinical trials. Anecdotally, it is reported that headache frequency varies from month to month in individuals with migraine, although the natural variation of an individual's headache frequency is not often considered in clinical or research contexts. The nature of headache frequency variation in the migraine population is largely unexplored. This study examined empirically derived, theoretically supported models of headache frequency changes in an individual with migraine over time by studying the American Registry for Migraine Research (ARMR) headache diary data.

The International Classification of Headache Disorders (ICHD)^1^ describes two frequency‐based diagnoses for migraine. Individuals with ≥15 headache days/month for >3 months, with ≥8 of the headache days being migraine days, are classified as having chronic migraine. Those with migraine not meeting these criteria are described as having episodic migraine.

Patients with chronic migraine have worse socioeconomic status, overall health‐related quality of life, and more health comorbidities than those with episodic migraine, in addition to the direct burdens due to increased headache frequency.^2^ Their condition is more likely to cause interpersonal and financial stress.^3^ The classification of an individual as having chronic migraine can have consequences for their treatment as some treatments are approved only for the treatment of chronic migraine, specifically onabotulinumtoxinA.^4^ Because the use of a 15‐headache days cutoff does not account for the natural variation in headache frequency, an individual can appear to vacillate frequently between chronic and episodic migraine^5^ when their underlying condition has not changed.

Moreover, recent research has suggested that three or four categories may be a more appropriate paradigm for understanding the relationship between headache burden and headache frequency.^7^

This study examined empirically derived models of headache frequency classes and changes in migraine over time using headache diary data from the ARMR.^8^ We investigated empirically supported frequency classes, frequency variability, and transition between frequency classes with the use of hidden Markov models (HMMs), which incorporate natural variability.

A Markov model is a probabilistic model that describes the movement of an entity among a series of states. In an HMM, the states are hidden and cannot be directly observed; only “emissions” from the states can be observed. By studying the emissions, we can learn about the hidden states and the transitions between states.

Each HMM is defined by the unobserved vector of initial state probabilities *π*_0_, the state transition matrix, and emission probability distributions. The initial state vector describes the probability of being in each state at time 0. The state transition matrix gives the probability of transitioning from each state to every other state at each time point. There is one emission probability distribution for each state; it describes the likely emissions from that state.

In our case, each state has a different headache frequency distribution, with the emissions being monthly headache frequencies. A person with migraine is the entity who exists in a state. While in a given state, they experience a number of monthly headache days determined by that state's emission distribution. From their current state, an individual may, in the next month, transition to a different state. The next state may have a larger or smaller expected number of headache days per month. The person may also remain in the same state while experiencing a varying number of headache days (determined by the emission distribution) each month.

## METHODS

### The ARMR

The ARMR was a multicenter prospective, longitudinal migraine patient registry^8^ that collected clinical data, electronic health record data, and neuroimaging data. Patients were invited to complete a headache diary and online longitudinal questionnaires detailing their headache characteristics and symptoms and psychological characteristics. The ARMR was funded by the American Migraine Foundation.

Upon enrollment, patients were asked about their sex and year of birth. In the online headache diary, patients were asked daily about headache occurrence, triggers, duration, severity, and its interference along with medication usage. Additional questionnaires covering headache features, interictal burden, sound and light sensitivity, and disability using the Migraine Disability Assessment Scale,^9^ pain interference (via the Patient‐Reported Outcomes Measurement Information System‐Pain Interference),^10^ skin sensitivity (12‐item Allodynia Symptom Checklist),^11^ sleep (custom), work productivity, and activity impairment (via the Work Productivity and Activity Impairment questionnaire)^12^ were available upon enrollment and at regular 3‐month intervals going forward.

### Standard protocol approval and patient consents

The ARMR participants were recruited and enrolled from eight ARMR sites using convenience sampling. The sites were: Mayo Clinic Arizona, Dartmouth‐Hitchcock Medical Center, University of Texas Health Science Center at Houston, University of Colorado, Georgetown University Hospital, Thomas Jefferson University, University of Utah, and DENT Neurological Institute. The ARMR was reviewed and approved by the Institutional Review Board at each site. Written informed consent was obtained from each participant prior to their participation in the study.

### Diary data preparation

The diary data were cleaned to remove duplicate entries. From there, they were aggregated across consecutive 28 calendar‐day “pseudo months,” with the first day of the first pseudo‐month beginning on the date of the patient's first diary entry. Aggregated data included the number of reported headache days per month, the number of completed diary entries per month by month, and the patient identifier.

There were significant missing data in the diary entries and with reason to believe that the data were not missing at random, incomplete data were dropped rather than imputed.

### Preliminary analysis

When examining the distribution of the reported headache frequency values, an apparently bimodal distribution was observed. This suggested headache frequency might be more accurately represented by a mixture distribution. Changes in headache frequency between all available within‐subject consecutive month pairs were calculated, and a transition matrix was created from the set of all consecutive month pairs. Examination of changes in headache frequency between sequential month entries showed that while some individuals experienced large swings in monthly headache frequency, most individuals' monthly headache frequency changes by only a few days from month to month.

### The HMMs

An HMM has a mixture distribution as its marginal distribution and preserves sequential dependency, meaning that while there is variation in output values, the next value depends partially on the current value, giving it the ability to limit the variability coming from a component distribution. With a multimodal distribution suggested by the headache frequency distribution and knowing that changes in headache frequency tend to be small, an HMM architecture was selected.

In the primary analysis, each headache frequency distribution was modeled with a Poisson distribution, as is common with count data, or with a constant value. All complete months of data from individuals with two or more complete months were utilized. Months did not need to be consecutive; non‐consecutive entries are accounted for in the fitting algorithm.

The optimal number of states was determined by fitting multiple models with varying numbers of states with and without sex as a covariate. Analysis was performed in R 4.2.3 with HMM models fit using the “msm” R package.^13^, ^14^

Each model is described by the states, which are defined by the parameters of their emission distribution, the transition matrix and initial state probability vector. The Akaike information criterion (AIC) was calculated for every model.

For each Poisson model, bootstrapped confidence intervals (CIs) on the rate parameter of each emission distribution and on the entries of the transition probability matrix were calculated by sampling subject identifiers with repetition and refitting the model 100 times.

To investigate the effect of the use of Poisson distributions, three HMM with truncated normal distributions and a fixed distribution and no covariates were fitted. Truncated normal distributions were limited to values between 0 and 28.

For comparison of intra‐subject variability, the observed data was randomly resampled for the same number of patients and number of observed months per patient as in the observed data to simulate a dataset with a matching overall distribution of monthly headache frequency but without individual serial dependence. For every month of data that a subject had in the historical data, a random draw was made from the pooled historical data. The intra‐subject median headache frequency and intra‐subject interquartile range of frequency were calculated, and the distribution of each was compared to the distribution of intra‐subject median headache frequency and interquartile range calculated from the historical data.

Similarly, each subject's headache frequency pattern was simulated using the best Poisson‐based model and separately with the best Truncated normal‐based model. The intra‐subject metrics were calculated and compared to the distribution of the intra‐subject metrics of the historical data.

The best model was selected based on complexity, AIC, and comparison of the intra‐subject distributions. To investigate the potential clinical meaning of the model states, we found the most likely state for each month of historical data via the Viterbi algorithm. We then paired each individual's ARMR quarterly questionnaire data with their closest available complete month of historical data. We performed a preliminary examination of the traits and symptoms using the fitted model state. Continuous variables were compared using the Kruskal–Wallis test, and categorical variables were compared with chi‐squared tests.

## RESULTS

Patients were enrolled into ARMR between February 2016 and April 2020. Of the ARMR patients with a migraine diagnosis, 981 completed at least one entry in the headache diary.

The original data set included 6432 pseudo‐months of data with at least one diary entry from 981 different patients. After limiting the dataset to complete months (pseudo‐months with 28 diary entries per 28 days) and patients with >1 complete month, there were 3407 months of data from 447 different patients (Table 1). In all, 378 (84.6%) of the individuals in the final data reported female sex. Approximate mean (standard deviation) age at enrollment was 50.4 (13.0) years. In all, 415 participants self‐reported White race; nine, Black or African American; four, Asian; three, American Indian or Alaska Native; two, Hawaiian or Other Pacific Islander; and the remaining reported “Unknown” or did not answer.

**TABLE 1:** Summary of data used to fit hidden Markov models.

Patients, *n*	Across patients, *n*, median (interquartile range)				Total months data, *n*
	Months with data/patient	Calendar span (28‐day months)	Monthly headache days/patient	Monthly diary entries/patient	
All data with 1+ entry/month					
981	4 (2–9)	5 (2–10)	10 (5–19)	27 (8.5–28)	6432
28‐diary‐entry months					
593	4 (2–8)	5 (2–9)	16 (9–28)	28 (28–28)	3643
28‐diary‐entry months and >1 month/patient					
447	6 (3–10)	6 (3–12)	16 (8–27.5)	28 (28–28)	3407

*Note*: Count of months with full or partial data in row 1, full data in subsequent rows.

On average, each participant had 7.6 months of data originally spanning 8.6 28‐day calendar periods, illustrating that while the selected entries were dense sequentially, the entries were not necessarily from consecutive periods.

### Preliminary transition analysis

**FIGURE 1:**
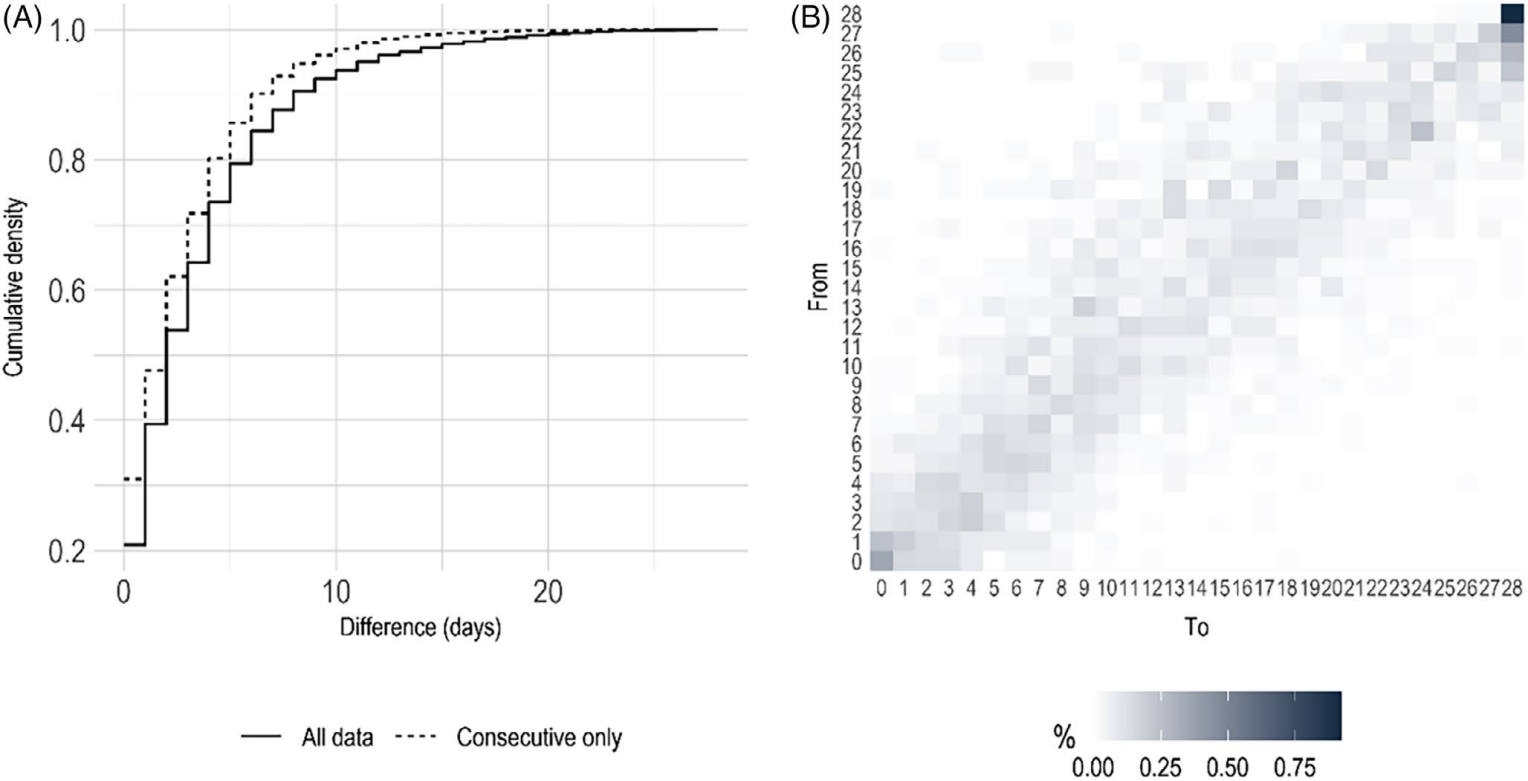
Month‐to‐month headache frequency changes. (A) Cumulative density for month‐to‐month differences in headache frequency in the entire diary data set and in the subset including only data with 2 consecutive complete months. (B) Monthly headache frequency transitions for the subset of consecutive months only as percentage of each origin frequency (row). [Colour figure can be viewed at wileyonlinelibrary.com]

In Figure 1A, it is seen that 75% of all months showed an absolute change in headache frequency of ≤5 days compared to the previous month. Figure 1B shows the transitions between sequential monthly headache frequencies in the set of complete diary months followed by another complete diary month. In this subset, the difference between two consecutive monthly headache counts was 0 or 1 48% of the time. Of transitions from a month with 28 headache days/month, 92% were to a month with 28 headache days/month.

In 23.9% of months in the final data set, the study participants reported experiencing a headache every day. In the subset of data from individuals with at least 1 month of daily headache, 73.0% of reported months had daily headache, indicating that those with a single month of daily headache have a high likelihood of subsequent months of daily headache. This finding suggested the inclusion of a daily headache state in the HMM.

### The HMM results

Results from the fitted HMMs are shown in Table 2. Fit metrics and 95% CIs on the rate parameters are shown.

**TABLE 2:** Summary statistics from Poisson‐based hidden Markov models.

*N* components	Sex	AIC	State, mean (95% confidence interval)			
			1	2	3	4
2	False	22,691	7.15 (6.99–7.32)	23.51 (23.24–23.78)		
2	True	22,695	7.15 (6.99–7.32)	23.5 (23.23–23.77)		
3	False	17,875	6.04 (5.84–6.26)	17.79 (17.43–18.16)	28 (28–28)	
3	True	17,900	5.99 (5.79–6.2)	17.73 (17.37–18.09)	28 (28–28)	
3	False	20,400	4.32 (4.1–4.56)	12.55 (12.16–12.96)	26.06 (25.7–26.43)	
3	True	20,346	4.36 (4.15–4.57)	12.72 (12.38–13.08)	26.23 (25.89–26.57)	
4	False	16,508	3.52 (3.24–3.82)	10.11 (9.62–10.61)	20.29 (19.78–20.82)	28 (28–28)
4	True	16,509	3.54 (3.29–3.81)	10.11 (9.66–10.59)	20.35 (19.84–20.87)	28 (28–28)
4	False	19,696	2.54 (2.32–2.78)	8.15 (7.81–8.5)	15.6 (15.07–16.15)	26.75 (26.41–27.09)

Abbreviations: AIC, Akaike Information Criteria; rates present means and 95% confidence intervals; Sex, was sex included as a covariate; *N* components, number of emission distributions/states.

Models including one constant distribution had a better fit than models with the same number of component distributions but with only Poisson distributions. Including sex as a covariate showed a slight improvement in fit over the same model without sex as a covariate only for the model with three Poisson states and no constant state. Sex was deemed not worth the additional model complexity. Model fit increased with the number of components. The size of the dataset was too small to fit models with additional components.

The model with three truncated normal distributions and one constant did have a very slightly improved AIC versus the comparable Poisson model when it converged, but it frequently failed to converge (Table 3). This is due to the additional parameters required by a truncated normal distribution, which increases the difficulty of fitting the model.

**TABLE 3:** Fit information for hidden Markov model with truncated normal distributions.

*N*	AIC	State			
		1	2	3	4
2	18,493	Tnorm (mean = 15.07, SD = 2701.32)	Constant 28		
3	16,788	Tnorm (mean = 5.71, SD = 4.79)	Tnorm (mean = 21.92, SD = 8.08)	Constant 28	
4	16,018	Tnorm (mean = 3.45, SD = 4.11)	Tnorm (mean = 13.12, SD = 4.4)	Tnorm (mean = 76, SD = 16.65)	Constant 28

*Note*: Sex not included as covariate. Truncated normal distributions bounded between 0 and 28.

Abbreviations: AIC, Akaike Information Criteria; SD, standard deviation; Tnorm, truncated normal.

### Comparison to random variability

**FIGURE 2:**
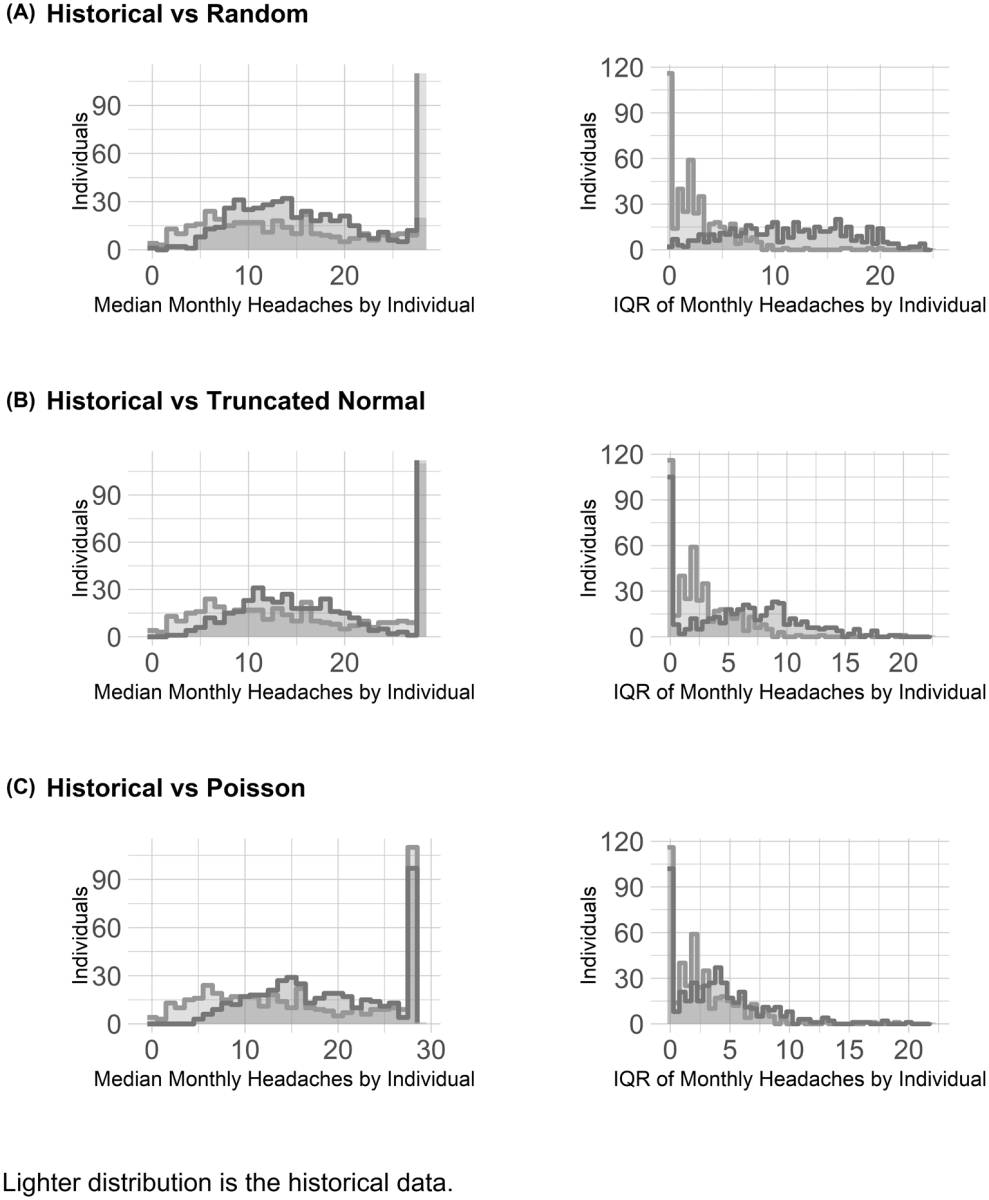
Comparison of distribution of individuals’ medians and individuals’ IQRs of monthly headache days: (A) Between observed historical data and samples generated by drawing from historical data; (B) between observed historical data and samples generated hidden Markov model (HMM) with three truncated normal distributions and one constant distribution; and (C) between observed historical data and samples generated from HMM with three Poisson and one constant distribution. IQR, interquartile range; Pois, Poisson; Trunc Norm, truncated normal.

The HMM allows but restricts variability in a way that results in individual median monthly headache frequency and intra‐individual variance closer to observed data. Modeling an individual's headache frequency as random draws from historical data creates over‐centralized individual median monthly headache frequency and intra‐individual variability in excess of that found in the observed data (Figure 2A).

Similarly, Figure 2B shows that the HMM with three truncated normal distributions results in excess variation in individual monthly headache frequency and median monthly headache frequency values that are under‐dispersed compared to the observed data and the Poisson HMM (Figure 2C).

The model with three Poisson and one constant emission distribution and without covariates was selected as the best, most parsimonious model for further evaluation. The rate parameters for the Poisson emission distributions for this model were: *λ*_1_ = 3.52 (95% CI 3.24–3.82), *λ*_2_ = 10.11 (95% CI 9.62–10.61), and *λ*_3_ = 20.29 (95% CI 19.78–20.82). The fourth state had constant emissions of 28 headache days/month.

### Four component Poisson HMM

**FIGURE 3:**
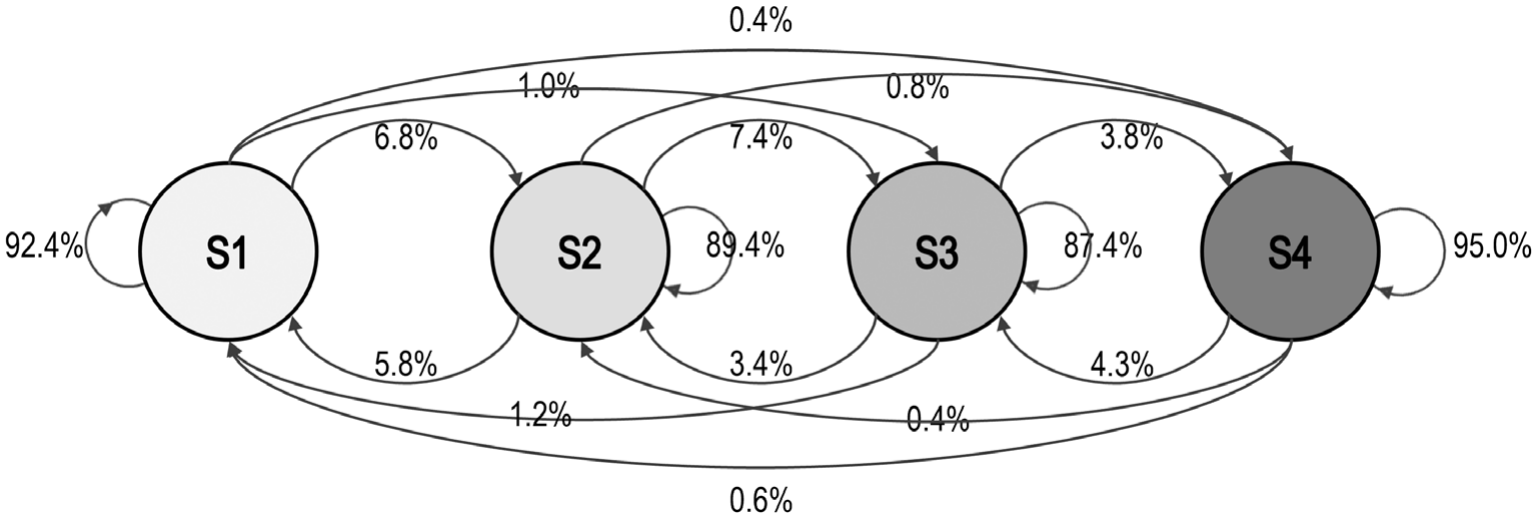
State transition diagram for model with three Poisson and one constant emission distribution/states. The value on each arc is the probability of transition.

Bootstrapped transition probability estimates and CIs are shown in Table 4, and the state transition diagram for the model is shown in Figure 3. The value of each arc is the probability of transition. They show that the most likely action from every state is to remain in that state with a decreasing likelihood of transitioning to “further” states (i.e., states with more divergent mean values).

**TABLE 4:** Transition probability matrix for model with three Poisson and one constant emission distributions.

From	To			
	1	2	3	4
1	0.92 (0.89–0.97)	0.06 (0.02–0.09)	0.01 (0–0.02)	0.01 (0–0.01)
2	0.07 (0.04–0.1)	0.89 (0.86–0.93)	0.03 (0.02–0.05)	0 (0–0.01)
3	0.01 (0–0.02)	0.07 (0.05–0.1)	0.87 (0.83–0.91)	0.04 (0.02–0.06)
4	0 (0–0.01)	0.01 (0–0.02)	0.04 (0.02–0.06)	0.95 (0.92–0.97)

*Note*: Bootstrapped mean transition probabilities (95% confidence interval).

**FIGURE 4:**
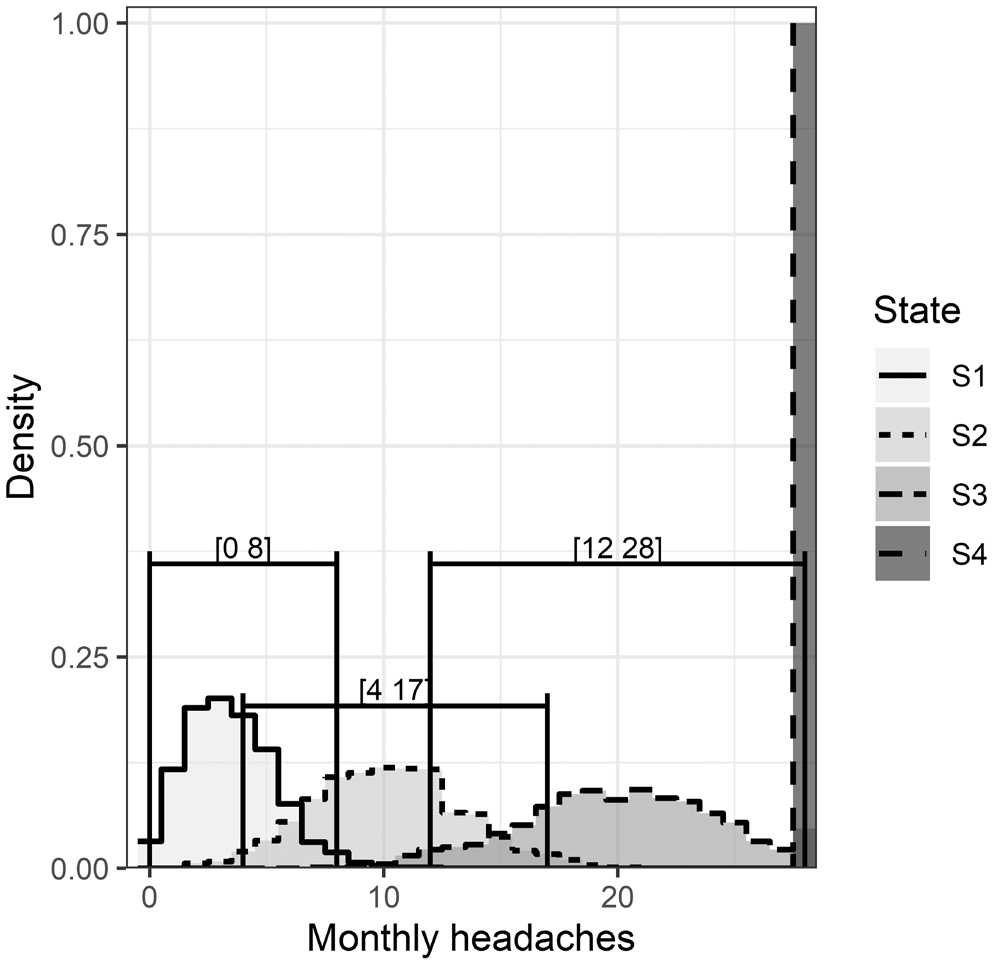
Illustration of the emission distributions. Samples generated from each emission distribution/state at the percentage equal to the initial state probabilities. 95% prediction intervals shown for the three Poisson distributions.

Simulated data from the four emissions distributions and corresponding 95% prediction interval (PI)s (State 1: 95% PI 0–8, State 2: 95% PI 4–17, State 3: 95% PI 12–28) are shown in Figure 4. There was a significant overlap in the 95% PIs. An observation of 15 headache days/month would not be exceptional from either the second or third state of this model, though it is most likely from the third state; 8.9% of measurements from the second state are expected to be ≥15 headache days/month and 9.4% of measurements from the third state are expected to be <15 headache days/month. With increased historical data, an individual with 15 headache days in 1 month could be more confidently placed into State 2 or State 3. The 80% PIs for the Poisson components (not shown) were: State 1: 80% PI 1–6, State 2: 80% PI 6–14, State 3: 80% PI15–26, and are nearly separated.

#### State interpretation

The results of the examination of traits and symptoms by likely HMM state are shown in Table 5.

**TABLE 5:** Headache trait and characteristic differences between fitted hidden Markov model states.

Headache‐related trait or characteristic	*p*
MIDAS total score	<0.001^b^
MIDAS pain scale	<0.001^b^
GAD total score category classification	<0.001^b^
Duration (category) of untreated headache	<0.001^a^
Type of headache pain when the pain is at its worst (throbbing, squeezing, tightness etc.)	0.509^a^
Frequency of …	
…nausea	<0.001^a^
…vomiting	0.061^a^
…dizziness/non‐vertigo	<0.001^a^
…vertigo	0.010^a^
…neck pain	0.002^a^
…light sensitivity	0.001^a^
…noise sensitivity	0.448^a^
…smell sensitivity	0.001^a^
…pain worse with routine physical activity	0.281^a^
…pain worse with routine mental activity	0.009^a^
…one‐sided weakness	0.074^a^
…seeing “shimmer lights, lines, dark spots, other shapes or colors”	0.006^a^
PROMIS pain interference *T*‐score	<0.001^b^
WPAI presenteeism	<0.001^b^
WPAI absenteeism	<0.001^b^
WPAI total work productivity impairment	<0.001^b^
WPAI total activity impairment	<0.001^b^

Abbreviations: GAD, Generalized Anxiety Disorder assessment; MIDAS, Migraine Disability Assessment Scale; PROMIS, Patient‐Reported Outcomes Measurement Information System; WPAI, Work Productivity and Activity Impairment questionnaire.

^a^ Chi‐squared.

^b^ Kruskall–Wallis test.

Many of these traits are known to vary with headache frequency, which is intimately connected with the HMM state, so these results should be interpreted accordingly. However, the analysis did suggest that the states have clinical interpretation.

## DISCUSSION

This analysis presents a potential method for clustering patients with migraine into meaningful groups by headache frequency while accounting for natural variation in frequency. Here, a model with four component distributions was found to be better than those with three or fewer components. The models with the fourth component distribution being constant were better than those with four Poisson distributions.

In this HMM, an entity—an individual with migraine—exists in a state. That state determines the range of headache frequency that an individual is likely to experience. At the end of each period, the entity may change state or remain the same. The model allows for variation in headache frequency even while the entity remains in the same state. In application, an individual's headache history could be collected, and their current state could be estimated. The current state will suggest a limited range of headache days the individual is likely to experience in the future. The current state can define useful and potentially clinically meaningful groups. Examination of the likely path through states based on historical data could indicate episodes in the past where the individual apparently changed state, increasing or decreasing headache frequency beyond natural variation.

In this model, the probability of having exactly 15 headache days/month is 3.7% for someone in the second state (*λ* = 10.1) and 4.8% for someone in the third state (*λ* = 20.34) of this model, suggesting this model has failed to bring clarity to the issue of a 15 headache days/month cutoff for chronic migraine. However, the chances of someone in the second state experiencing ≥15 headache days/month is only 8.9%, while for someone in the third state, this probability is 90.6%. Individuals in either state may experience 15 headache days/month but an individual in the third state is more likely to have ≥15 headache days in subsequent months, more closely aligned with a chronic migraine diagnosis.

### Transitions to and from chronic migraine

In the final data set of complete non‐consecutive months, 12.0% of sequential transitions were across the 15‐headache days chronic migraine cutoff, while 38.7% of those also involved a change in the fitted HMM state. When each individual's first month was excluded, creating more stable state estimates, those percentages changed to 11.6% and 28.2%, respectively. This demonstrates how an HMM may be a more stable classification method that accounts for individual variation while still maintaining frequency‐based classes.

A recent paper by Ishii et al.^7^ showed that headache burden and disability differed widely across headache frequency and that the binary chronic/episodic breakdown was insufficient to describe the differences. They evaluated headache burden and disability across four non‐overlapping groups: Group 1: 0–7 headache days/month, Group 2: 8–14 headache days/month, Group 3: 15–23 headache days/month, and Group 4: ≥24 headache days/month. The analysis found that the Migraine Disability Assessment Scale score but not the Patient‐Reported Outcomes Measurement Information System‐Pain Interference T‐score or Work Productivity and Activity Impairment questionnaire score differed between Group 2 and Group 3 and interpreted this as evidence that the 15‐headache days cutoff for chronic migraine was insufficient and suggested including Group 2 under the umbrella of chronic migraine.

In this work, the 80% PIs for the Poisson distributions ([1–6], [6–14], [15–26]) were nearly separated and largely align with the categories evaluated in Ishii et al.^7^ except at the very high frequency end, generally supporting the recommendations of Ishii et al.^7^

Fischer‐Schulte and May^15^ have described five concerns with suggestions to alter the ICHD criteria in response to Ishii et al.^7^ Three of those concerns would be addressed by this model. The first point was “one must draw the line somewhere.” This model preserves classification by headache frequency without drawing a hard line. The overlapping emission distributions allow for variation without changing state, even around the current 15‐headache days episodic/chronic migraine cutoff. Second, Fischer‐Schulte and May^15^ called for a data‐driven approach, which this is. Finally, this model does account for small month‐to‐month changes in headache frequency and larger changes in frequency over time (point four).

In a secondary analysis of the Chronic Migraine Epidemiology and Outcomes (CaMEO) study, it was observed that of individuals diagnosed with chronic migraine, only 26.6% met the ≥15 headache days/month diagnostic criteria for chronic migraine each of four or five waves of data, with waves collected every 3 months. The other 73.4% had episodic migraine in at least one of those periods. Of those with episodic migraine at baseline, 7.6% met the ≥15 headache days/month cutoff at least once.^5^ The tendency of individuals to round their headache frequency to multiples of five^6^ also diminishes the reliability of the 15‐headache days cutoff. The approach presented here may be useful in addressing these issues.

In this study, 71.5% of those with ≥15 headache days/month in the first period and 57.6% of individuals who had ≥15 headache days/month in any period had ≥15 headache days/month in each period, though the number of available months of data per individual varied in our study. This difference in findings is likely explained by the difference in the study population and the instrument used to measure headache frequency. This study population featured a large percentage of individuals with a daily headache. Among those with high but not daily headache frequency (15–27 headache days/month) in the first month, 48.6% had at least 1 month with <15 headache days/month.

Another recent study of headache diary data found that 50.7% of study participants analyzed had a change in episodic/chronic status based on <15/≥15 headache diary days after 12 months.^16^ The authors found that having a cyclic phenotype was associated with a greater change in headache frequency from the first to the last month. Modeling with HMM allows for cyclic behavior while also permitting changes that are not cyclic. Research on cyclic patterns in migraine could be used to guide decisions about the selection of horizon length for higher‐order HMM.

This work suggests the value of considering patients with daily headache as a separate population from those with high‐frequency migraine. Individuals who recorded at least one month of daily headache were more likely to have the same number each month, with over half of those with at least one entry of daily headache having daily headache each recorded month.

This work suggests a path for a more objective evaluation of migraine frequency progression as a shift from one state to another would represent a meaningful change in frequency rather than a natural variation in a patient's norm. A patient's historical headache frequency data could be used to estimate which state they are currently occupying and correspondingly (i) the number of headache days they are likely to experience, (ii) the likelihood of shift to a different state, and (iii) to establish if an apparent shift in frequency is a true shift or natural variation. This is potentially relevant to migraine clinical trials.

This approach may also be helpful for the identification of secondary headache in those with migraine. The ICHD has been moving towards more objective criteria for diagnosing secondary headache in the presence of pre‐existing primary headache, but room for subjective interpretation of the “made *significantly worse*” criteria still exist. The approach presented here may be useful in identifying meaningful shifts that indicate secondary headache onset or resolution. This is particularly relevant in the context of post‐traumatic headache in those with migraine, as post‐traumatic headache frequently presents with a migraine phenotype.^17^

From a research perspective, the component distributions suggest potentially clinically meaningful subgroups for further evaluation.

## LIMITATIONS

As described, the ARMR patient population^8^ was recruited from headache specialty clinics. It is likely this population is not representative of the general population with migraine from a headache frequency perspective as well as in terms of racial, ethnic, and other demographic categories. Patients at headache specialty clinics may have a higher headache frequency than the general migraine population. Headache days were used instead of migraine days because the diary did not collect information on headache characteristics that would have been needed to determine if the headache was a migraine.

This analysis only used data from months with 28 diary entries from the ARMR participants with >1 month of 28 diary entries per month. This is a small portion of the available diary entries. However, there is reason to believe that patients are more likely to complete the diary on days they experience headache than on days without headache, making it difficult to impute missing data accurately.

As with the potential bias in headache frequency due to the study population, this issue is most likely to affect the initial state probabilities, and these issues would affect parameter estimates. It is not expected to affect the validity of the approach. This analysis fit a range of HMMs with Poisson and truncated normal distributions. Many other distributions and combinations of distributions could be considered. The number of components considered here was limited by the size of the dataset but could be increased if a sufficiently large dataset was used. The models used here were first order HMMs, but higher order models are also possible.

Sex was considered as a covariate and found to only minimally improve the model; other variables may be more relevant. Medication use was not included in the model. Of individuals with at least one diary entry, 91.2% (1277/1400) reported using a medication on at least 1 day. Of pseudo‐months, ~39% recorded differences in acute or preventive treatments or both compared to the prior month. This high rate of medication change may be accurate, or it may be the result of incomplete or inaccurate diary information, as entering drug information was more time‐consuming and complicated than indicating the presence or absence of a headache. The frequency of medication usage and medication changes, and the wide variety of medications and treatments utilized make explicitly modeling medication consumption very difficult. A preliminary analysis did not show an association with modeled change between states and reported preventive or acute medication changes. The lack of association between particularly preventive medication changes and state changes may reflect inaccuracy in the medication information, or it may reflect that preventive medications are frequently ineffective or take time to become effective. In the final dataset in months where a new preventive was introduced, headache frequency increased slightly compared to the previous month. By failing to account explicitly for effective preventive treatment in the model, it may inflate the state‐to‐state transition probabilities.

To model natural variations and state changes it would be ideal to collect similar data on a population of individuals naïve to treatment.

Prior to making recommendations based on this approach, this model should be validated by completing a similar analysis in another population, preferably using data collected for this purpose. It needs to be seen whether these state definitions hold true in populations of individuals with migraine outside of headache specialty centers. It also remains to be verified whether state changes are associated with effective migraine prevention therapy.

## CONCLUSION

Our data showed that a large percentage of subjects reported headache every day. We also found that the month‐to‐month change in headache frequency tended to be small and not infrequently crossed the 15‐headache day chronic migraine cutoff.

This study used a semi‐unsupervised approach to finding natural subpopulations based on headache frequency and modeling the variability in headache frequency in those with migraine.

This study identified an HMM with three states with Poisson (rates: 3.52, 10.119, 20.29 headache days/month) emission distributions and an additional state with a constant emission of 28 as the best of the models considered based on AIC. Each of these four states describes a headache frequency‐based subpopulation with the specified mean headache days per month. States three and four most closely match the current chronic migraine definition but the HMM model allows variation about those mean values without any hard cutoffs. This model provided more stable group classification than a 15‐headache day cutoff. After further validation, HMM derived subpopulations may define useful diagnostic categories and an alternative to the current hardline chronic migraine, episodic migraine categories.

It has been repeatedly shown that there are meaningful differences between those with more or less frequent migraine. It has not been shown that the current cutoff is the optimal cutoff or that a hard cutoff is the best approach for categorizing patients with migraine. This paper does not recommend adoption of the specific model presented here, but rather it presents an argument for the new approach and a suggestion of what a specific model with this approach may look like.

## AUTHOR CONTRIBUTIONS

**Gina M. Dumkrieger:** Conceptualization; data curation; formal analysis; methodology; writing – original draft. **Ryotaro Ishii:** Conceptualization; methodology; writing – review and editing. **Peter J. Goadsby:** Conceptualization; methodology; writing – review and editing.

## FUNDING INFORMATION

The ARMR registry was supported by the American Migraine Foundation. No financial support was provided specifically for this study.

## CONFLICT OF INTEREST STATEMENT

**Gina M. Dumkrieger** has received research support from Amgen, the American Brain Foundation and the American Academy of Neurology. **Ryotaro Ishii** has served as a consultant for Amgen K.K., Eli Lilly Japan K.K., Daiichi Sankyo Company, Limited, and Otsuka Pharmaceutical Co., Ltd. He has received lecture fees from Amgen K.K., Eli Lilly Japan K.K., Daiichi Sankyo Company, Limited, and Otsuka Pharmaceutical Co., Ltd. **Peter J. Goadsby** reports, over the last 36 months, grants and personal fees from Eli‐Lilly and Company, grant from Celgene, and personal fees from Eon Biopharma, Allergan/Abbvie, Biohaven Pharmaceuticals Inc., CoolTech LLC, Dr Reddys, Epalex, Impel Neuropharma, Lundbeck, Novartis, Praxis, Sanofi, Satsuma and Teva Pharmaceuticals.

## References

[1] Headache Classification Committee of the International Headache Society . The International Classification of Headache Disorders, 3rd edition. Cephalalgia. 2018;38(1):1‐211. 10.1177/0333102417738202 29368949

[2] Manack AN , Buse DC , Lipton RB . Chronic migraine: epidemiology and disease burden. Curr Pain Headache Rep. 2011;15(1):70‐78. 21063918 10.1007/s11916-010-0157-z

[3] Buse DC , Fanning KM , Reed ML , et al. Life with migraine: effects on relationships, career, and finances from the Chronic Migraine Epidemiology and Outcomes (CaMEO) study. Headache. 2019;59(8):1286‐1299. 31407321 10.1111/head.13613 PMC6771487

[4] Dodick DT , Catherine C , DeGryse RE , et al. OnabotulinumtoxinA for treatment of chronic migraine: pooled results from the double‐blind, randomized, placebo‐controlled phases of the PREEMPT clinical program. Headache. 2010;50(6):921‐936. 20487038 10.1111/j.1526-4610.2010.01678.x

[5] Serrano D , Lipton RB , Scher AI , et al. Fluctuations in episodic and chronic migraine status over the course of 1 year: implications for diagnosis, treatment and clinical trial design. J Headache Pain. 2017;18(1):1‐12. 28980171 10.1186/s10194-017-0787-1 PMC5628086

[6] Houle TT , Turner DP , Houle TA , et al. Rounding behavior in the reporting of headache frequency complicates headache chronification research. Headache. 2013;53(6):908‐919. 23721238 10.1111/head.12126 PMC4546843

[7] Ishii R , Schwedt TJ , Dumkrieger G , et al. Chronic versus episodic migraine: the 15‐day threshold does not adequately reflect substantial differences in disability across the full spectrum of headache frequency. Headache. 2021;61(7):992‐1003. 34081791 10.1111/head.14154

[8] Schwedt TJ , Digre K , Tepper SJ , et al. The American registry for migraine research: research methods and baseline data for an initial patient cohort. Headache. 2020;60(2):337‐347. 31755111 10.1111/head.13688

[9] Stewart WF , Lipton RB , Kolodner KB , Sawyer J , Lee C , Liberman JN . Validity of the Migraine Disability Assessment (MIDAS) score in comparison to a diary‐based measure in a population sample of migraine sufferers. Pain. 2000;88(1):41‐52. 11098098 10.1016/S0304-3959(00)00305-5

[10] Cella D , Riley W , Stone A , et al. The Patient‐Reported Outcomes Measurement Information System (PROMIS) developed and tested its first wave of adult self‐reported health outcome item banks: 2005–2008. Clin Epidemiol. 2010;63:1179‐1194. 10.1016/j.jclinepi.2010.04.011 PMC2965562 20685078

[11] Bigal ME , Ashina S , Burstein R , et al. Prevalence and characteristics of allodynia in headache sufferers: a population study. Neurology. 2008;70(17):1525‐1533. 18427069 10.1212/01.wnl.0000310645.31020.b1 PMC2664547

[12] Reilly M , Zbrozek A , Dukes E . The validity and reproducibility of a work productivity and activity impairment instrument. Parmacoeconomics. 1993;4(5):353‐365. 10.2165/00019053-199304050-00006 10146874

[13] R Core Team . R: A language and environment for statistical computing. R Foundation for Statistical Computing; 2022.

[14] Jackson CH . Multi‐state models for panel data: the msm package for R. J Stat Softw. 2011;38(8):1‐29.

[15] Fischer‐Schulte L , May A . The 15‐day threshold in the definition of chronic migraine is reasonable and sufficient‐five reasons for not changing the ICHD‐3 definition. Headache. 2022;62:1231‐1233. 36286599 10.1111/head.14383

[16] Gallardo VJ , Alpuente A , Pozo‐Rosich P . Association of a cyclical migraine phenotype with disease progression: a 1‐year time series analysis. Neurology. 2022;99(12):e1326‐e1334. 35953289 10.1212/WNL.0000000000200887

[17] Lucas S , Hoffman JM , Bell KR , Dikmen S . A prospective study of prevalence and characterization of headache following mild traumatic brain injury. Cephalalgia. 2014;34(2):93‐102. 23921798 10.1177/0333102413499645

